# Atlanta Society of Medicine

**Published:** 1889-05

**Authors:** 


### ATLANTA SOCIETY OF MEDICINE.

### A PLEA FOR THE MEATUS URINARIUS.

MEETING MARCH 19, 1889.

President Hunter P. Cooper, M. D., in the chair.

Dr. Olmsted read a paper, "a plea for the meatus urinarius," in which he said, that it was a fashion now-a-days, to slit up the meatus for any and every condition that may happen to exist. That the operation was very unskillfully performed for the most part, and that it was an injustice to Dr. Otis, (who had done more than any one else of the present time, for the relief of these urethral troubles,) to allow these gaping meati to stand as a result of "an operation after Otis." That it was not true, he did not suggest, or recommend any such. Dr. Olmsted said that while he had great respect for the teachings of Dr. Otis, he was compelled to take issue with him with regard to the normal meatus. Dr. Otis did not hold that the meatus was narrower than the canal behind it, also, that he (Dr. Otis) did not hold that the fossa navicularis was a normal condition. Dr. Olmsted said, that his observations lead him to consider the meatus narrower than the canal behind it, and that he was sure that nature thoroughly demonstrated the fact, and spoke of the hose and nozzle as an illustration. The urine being expelled from the meatus in the same manner that water was driven through the nozzle of a hose pipe. Dr. Olmsted said that he held that the fossa navicularis was a normal anatomical condition. The fact that Dr. Brown could not find it present in the newly born infant, was no proof that it did not exist in the adult. Dr. Brown, of Baltimore, the authority whom Dr. Otis quoted as not being able to find the fossa navicularis in infants, himself acknowledges that the meatus is narrower than the canal behind it. The paper was very full and interesting and is published in full on page 129.

Dr. Elkin, opening the discussion, said that he had enjoyed Dr. Olmsted's paper very much indeed. That it was instructive, and very comprehensive. That there was one point that was worthy of our consideration, viz.: the normal meatus. A normal meatus, Dr. Elkin defined, as one that occasioned the person no inconvenience, be it small or large, the fact of its not creating any trouble, nervous or otherwise, was the indication of its being normal, the size having nothing to do with it. In performing the operation of slitting the meatus, care should be taken; sometimes very serious conditions resulted from a bungling operation, such as hemorrhages to an excessive amount, and even death had occurred. The slit ought to be no larger than necessary, and ought to be made on the floor of the meatus. That he considered it very unfortunate to be compelled to slit the meatus above, but that *this* is required in some instances, in order to avoid a hypospadic condition. He thought that all strictures, within an inch of the meatus, should be divided at the same time the meatus was operated upon, and consequently remove any further source of trouble, and insure benefit. He was of the opinion that the operation was abused, and that many cases were operated upon, when there was no need of any such interference.

Dr. McRea said, that he had enjoyed Dr. Olmsted's paper very much. That he had had but comparatively little experience as compared with some others, but that he thought that the meatus was often cut when there was no occasion for it. When he had to perform the operation, he cut both above and below, and was of the opinion that he had a better result. Had a case of a young man whose meatus was small and apparently normal, but on examination, the urethral slit was found to be but superficial, and the true urethral orifice to be under the floor in a kind of pouch. This he remedied by an incision above and below. Said it was impossible to prevent hypospadia after the operation.

Dr. Calhoun said, that he regretted not hearing the first part of Dr. Olmsted's excellent paper. That he had but little experience with such cases, but taking a common sense view of the question, he would conclude that the cutting should not be very extensive. That the operation of slitting the meatus urinarius, was analogous to the operation of slitting up the lachrymal sack, which, if opened too much, gives the patient a great deal of inconvenience. He would judge that too large a slitting of the meatus urinarius would entail a great amount of inconvenience and suffering upon the patients so treated.

Dr. Stockton called attention to a fact that was often overlooked, viz : that there was a circular band of muscular fibres around the meatus urinarius, a kind of sphincter, and that *this* aided the expulsion of the last drops of the urine, and was easily dilated by the urine, propelled by the vis-a-tergo powers inherent to the parts. There was no need of destroying it entirely with the ruthless knife, and occasioning the patient so much discomfort.

Dr. Bak spoke of the need of cleanliness and saw no occasion for the use of the knife in every case. Mentioned cases which had been brought to him with a request that he operate, but after examination and treatment, other than operation, he had demonstrated the fact to the patients, that operation was not at all necessary. Dr. Bak said also, that he was sorry that his coming late had lost him the pleasure of hearing the major part of Dr. Olmsted's very interesting paper.

Dr. Cooper expressed himself as being very much pleased indeed with Dr. Olmsted's paper. Said he was not an advocate of meatotomy for all small meati. Nor did he believe in circumcision, to the extent that it was now carried. He did not think that it was intended that men should be deprived of their foreskins for trivial conditions. Dr. Cooper, in speaking of the extensive slitting of the meatus, related the case of a man who had had his meatus largely slit, and who suffered misery from having the lips slip on each other, until one lip projecting beyond the other exposed the mucous membrane, and this membrane coming in contact with his flannel drawers, caused irritation and reflex nervous disturbance, more grave than those for the relief of which the operation had been performed. He had also noticed that the slitting had failed to reach and relieve the stricture behind the meatus, and that although the meatus was made to represent a yawning chasm, the patient was in no wise benefitted by the operation so performed.

Dr. Olmsted, in closing the discussion, said that he was glad to see that all who had spoken on the subject, were of one mind. That he differed with Dr. Otis in this only, that he held that the meatus was narrower than the canal behind it. Dr. Otis did not, but so far as the operation as suggested by Dr. Otis was concerned, and as performed by him, he had this to say: that if performed as Dr. Otis performed it, there would be no gaping or hypospadias. He took issue with Dr. Otis only as to the anatomical condition of the normal meatus. The conical shape of the glands suggested the narrow condition of meatus as intended by nature. In conformity with Otis' law relative to the proportional diameters of the urethra and penis the enlargement in fossa navicularis should correspond with the greater diameter of the glands, and the tapering of the glands indicate the corresponding narrowing of the meatus. He further said, that in operating, one should follow closely after nature, and be sure that the operation was made to meet the indications and not the indications to meet the operation. Many times the operator did not reach the point which needed division, consequently did not relieve the patient, but left him with a wide, gaping meatus, which caused his urine to sprazzle and his condition to be a very pitiable one. He said also, that the point made by Dr. Elkin, that the normal meatus was one that did not give the party any uneasiness was a good one and would serve as a guide. The point made by Dr. Stockton was also very excellent, and would serve to explain in part, the narrowness of the meatus and as it was dilatable, it was not therefore necessary to cut to such an extent as is now the fashion. He concluded by saying that the meatus was not always at fault, and that he hoped that the indiscriminate slitting of this tender portion of the human organism would cease to be so unnecessarily common, and that surgeons would be more accurate in their diagnosis, and that when the operation was necessary, that it would be more properly and skillfully performed, and the accident so timely mentioned by Dr. Cooper, would not occur to annoy their patients, or bring discredit upon their work.

---

MEETING, April 2d, 1889.

V. O. Hardon, M. D., in the chair.

PNEUMONIA.

Dr. Duncan reported a case of pneumonia. Said he was called to see a negro girl aged 15 years; found the upper lobes of the right lung very much affected. Temperature, 105; pulse, 120; respiration, 46; left lung not involved. For a few days, owing to poor nursing, she grew worse. Pulse, 130; temperature, 105; respiration, 79; continued to breathe fast for forty-eight hours, but eventually recovered. Said it was very unusual to have the upper lobes of the lung so extensively invaded, as in this case.

Dr. Van Goidtsnoven reported a case of bloody discharge from the vagina of a child seven days old. Said there was no lesion that he could find about the vagina; had at first thought that the parts had been injured by a diaper pin, but found that such was not the fact; the discharge continued for three weeks and the ceased. His treatment was directed only to keeping the parts clean. Dr. Van Goidtsnoven also reported a case of menstruation in a child four years old. Said he was called to see a girl four years old, who was suffering from intense headaches with discharge from vagina, and that her mother told him that the child had had her menses before, and had headache at the time, and that she supposed that that was the cause of the severe headache. He prescribed for the headache, but did nothing more.

Dr. Olmsted spoke of a case where menstruation had taken place in a child two years old. Said that he also had had a case of bloody discharge from the vagina of an infant, which he thought was caused by irritation of the lochial fluid.

Dr. Hardon mentioned a case as reported by Dr. T. G. Thomas, New York, of a child menstruating at the age of two years; that the mammae were enlarged, and that the same child became pregnant at the age of eight.

### HYSTERICAL APHONIA.

Dr. Calhoun reported five cases of hysterical aphonia, four of which were females, and one male; of the females, one only was married. All the symptoms were typical. On examination with the laryingoscope, nothing of a diseased nature was found. There was simply a lack of phonating power. The cords were in every respect natural in all the cases; it was purely a nervous affection. One of the ladies who was married, had had several accidents, and considerable family t·ouble. And one of those unmarried had suffered from uterine disease. Said the peculiarity of all the cases, was the irregularity of the attacks; also, that most of the patients had been affected for some length of time; also, that the dread of having an attack would bring on one. In the matter of treatment, Dr. Calhoun said, that a great desideratum was to have the patients understand that the affection was purely nervous, and that there was no organic lesion. This, with a tonic treatment, and the application of the galvanic current to the throat externally, would meet all the indications, and if persisted in, would permanently cure all such cases. Said that he sometimes applied the current to the cords, but that he did not believe there was any occasion to do so, for there was nothing like paralysis of the cords present, consequently the cords themselves needed no stimulation. Dr. Calhoun also said, that he had come to the conclusion, that the electricity acted as much mentally, as it did physically, that is, that its application seemed to produce as much of a tonic effect upon the minds of the patient, as it did upon the muscles, etc., of the throat.

WIDE MEATUS URINARIUS.

Dr. Olmsted reported a case of wide meatus, which he thought could be taken as an example of the condition of the meatus as claimed by Dr. Otis, to always exist, viz: as wide as the canal behind it—said that when he first examined this particular meatus, that it appeared to have been operated upon, but looking closely, he found that such was not the case. The patient had a stricture about one-fourth of an inch behind the meatus, which he divided. He said, the coming of this patient to him, would seem like a judgment for his having disagreed with that distinguished teacher, Dr. Otis, but that he could not, even now, believe that the meatus was as wide as the canal behind it.

DOUBLE MEATUS.

Dr. Elkin reported a case of double meatus, said that the patient presented a meatus, with a transverse slit across the verticle slit. That the partition was a semi-cartilaginous substance, which extended back for three-quarters of an inch. The patient had had no trouble with this condition of the meatus, in fact, did not know anything was abnormal, although he had always noticed a double stream when urinating. On contracting gonorrhœa, and trying to use an injection, he found that it would flow out as fast as he injected it, and had accordingly become alarmed, and called upon Dr. Elkin for relief. Dr. Elkin further said that he divided the septum, and that the patient was using an injection very successfully. Dr. Elkin also reported a case of urethral chancre, which had been masked by chancroids of the prepuce and glands penis. The patient complained of a stinging sensation near the meatus, and on feeling the penis at point of stinging sensation, felt a hard mass, which he concluded to be a chancre, and which was very thoroughly demonstrated by the usual secondary eruption making its appearance sometime later.

Dr. Olmsted spoke of having seen the meatus divided by a partition like the one mentioned by Dr. Elkin, and also remarked that in Quain's Anatomy, new edition, this partition was spoken of as the cloak of the lacuna magna.

OV RIOTOMY.

Dr. Hardon reported case of ovariotomy; said the patient was forty-three years old, and up to two years ago was well. About that time she noticed an enlargement of the right side of the abdomen, which continued to increase, and the usual impairment of the general health which accompanies such growths was present. He made an incision in the median line, and after tapping the cyst, drew it out through the incision, but finding that it was firmly bound to the pelvic organs by adhesions he did not make a pedicle, but drew the sack closely up to the abdominal walls, and cut it off; after having washed out the abdominal cavity with water that had been boiled, he stitched the walls of the cyst to edges of the incision, this left a pocket about an inch in depth, formed entirely of the cyst walls. Dressed the wound with antiseptics, and antiseptic gauze with a flannel bandage over all. Dr. Hardon said the fluid of the cyst was of a chocolate color, and that the fluid and cyst weighed thirty-seven and a half pounds; said he found no adhesion at upper part of the cyst. That the patient passed bloody urine for forty-eight hours after the operation, which he could not account for, except upon the supposition that the bladder was slightly injured during the operation. The temperature at no time exceeded 100.05, that there had been no inflammation around the edges of the wound, and that the pouch had granulated rapidly, and was nearly healed, and that the patient was sitting up. Dr. Hardon attributed the excellent results to the strict antiseptic precaution taken by him during the operation.

Dr. Calhoun said that with regard to the use of antiseptics, he took much pleasure in announcing the fact that in one hundred and nine operations for cataract, under antiseptic conditions, and with the free use of the bi-chlorid, all the cases had terminated without one particle of suppuration. Previous to his using antiseptics, as he now did, he had had bad results from suppuration of the cornea. His present plan was to wash the face, forehead and eyebrows, with castile soap and water, (after washing his hands) he did this washing himself. Then he washed the eyelids, both inside and outside, and the conjunctiva with bi-chloride solution, 1 to 10,000. Said he was astonished to see how well the conjunctiva stood the amount of scrubbing he gave it. He then flooded the cavity of the lids, and kept it flooded during the operation with the bi-chloride, solution 1 to 10,000, and did this also at every subsequent dressing. Used borated cotton as a covering, preferred it to the bi-choride gauze. Dr. Calhoun said that he used cocaine as a local anasthetic, one fourth of one per cent solution· That this solution was very effective. Some two years ago he used a much stronger solution of cocaine, and had bad results. He published a paper on the subject of strong solutions of cocaine being baneful in operations of the eye, and was very severely criticised by his friends North, and he had about concluded that he was the only one who had such an experience, when he received a report from a London Hospital detailing exactly a similar experience, and on visiting the New York hospitals (eye) he found that they also had like results from strong solutions of cocaine, but had not published them. Since using this one-fourth of one per cent solution, he had no trouble whatever, and that it was sufficient to thoroughly anæsthetize the parts, and prevent pain during the operation. In conclusion, Dr. Calhoun said that cocaine in strong solution contracted the blood-vessels of the eye, and that when the contracting influence wore off, the blood rushed back into the vessels and flooded the tissues with blood, inducing inflammation of a high degree, which resulted in sloughing of the cornea, etc., also that whiskey was the best remedy for the sinking feeling consequent upon absorption of excessive amount of cocaine.

